# Catastrophic health expenditure and its association with socioeconomic status in China: evidence from the 2011-2018 China Health and Retirement Longitudinal Study

**DOI:** 10.1186/s12939-023-02008-z

**Published:** 2023-09-21

**Authors:** Xi Li, Itismita Mohanty, Tiemin Zhai, Peipei Chai, Theo Niyonsenga

**Affiliations:** 1 Health Research Institute, Faculty of Health, University of Canberra, Building 23, 26 University Drive Street, Bruce, Canberra, 2617 Australia; 2 Department of Health Economics and National Health Accounts Research, China National Health Development Research Center, Beijing, China

**Keywords:** Socioeconomic status, Catastrophic health expenditure, Variable thresholds, Non-communicable diseases, Longitudinal study, China

## Abstract

**Background:**

An increase in healthcare utilization in response to universal health coverage may leave massive economic burden on individuals and households. Identifying catastrophic health expenditure helps us understand such burden. This study aims to examine the incidence of catastrophic health expenditure at various thresholds, explore its trend over years, and investigate whether it varies across socioeconomic status (SES).

**Methods:**

Data used in this study were from four waves of the China Health and Retirement Longitudinal Study (CHARLS): 2011, 2013, 2015, and 2018. SES was measured by annual per-capita household expenditure, which was then divided into quintiles (Quintile 1 (Q1): the poorest - Quintile 5 (Q5): the wealthiest). Catastrophic health expenditure was measured at both a fixed threshold (40%) and a set of variable thresholds, where the thresholds for other quintiles were estimated by multiplying 40% by the ratio of average food expenditure in certain quintile to that in the index quintile. Multilevel mixed-effects logistic regression models were used to analyze the determinants of catastrophic health expenditure at various thresholds.

**Results:**

A total of 6,953 households were included in our study. The incidence of catastrophic health expenditure varied across the thresholds set. At a fixed threshold, 10.90%, 9.46%, 13.23%, or 24.75% of households incurred catastrophic health expenditure in 2011, 2013, 2015, and 2018, respectively, which were generally lower than those at variable thresholds. Catastrophic health expenditure often decreased from 2011 to 2013, and an increasing trend occurred afterwards. Compared to households in Q5, those in lower quintiles were more likely to suffer catastrophic health expenditure, irrespective of the thresholds set. Similarly, having chronic diseases and healthcare utilization increased the odds of catastrophic health expenditure.

**Conclusions:**

The financial protection against catastrophic health expenditure shocks remains a challenge in China, especially for the low-SES and those with chronic diseases. Concerted efforts are needed to further expand health insurance coverage across breadth, depth, and height, optimize health financing mechanism, redesign cost-sharing arrangements and provider payment methods, and develop more efficient expenditure control strategies.

**Supplementary Information:**

The online version contains supplementary material available at 10.1186/s12939-023-02008-z.

## Background

Universal health coverage (UHC) aims to provide everyone with quality health services they need, without experiencing any financial hardship [1]. With the expansion of health service coverage, an increasing number of individuals tend to utilize health services to maintain better health and wellbeing [2]. However, the gains in service coverage may come at a major cost to individuals and their families [2–4]. Due to financial barriers to health access and health out-of-pocket payment, inadequate financial protection through health insurance coverage may leave a massive economic burden [5]. Globally, mitigating financial burden is a priority, reflected in its presence in the Sustainable Development Goals (SDGs) [3, 5, 6]. Achieving UHC (SDG Target 3.8) may facilitate alleviating financial barriers in case of illness [4].

Catastrophic health expenditure is often used for monitoring the global progress towards UHC [2, 6]. Examining catastrophic health expenditure is considered a critical component to understand the economic burden of health spending. Catastrophic health expenditure is defined as out-of-pocket payments on healthcare surpassing a predefined threshold of a household’s ability to pay [6]. Out-of-pocket payments are an important source of health financing across the globe [3], particularly in low- and middle-income countries (LMICs) where national health systems are underdeveloped [3, 4]. Out-of-pocket payments often account for as high as 40% of total medical expenditure in LMICs [3]. The occurrence of catastrophic health expenditure may impede people’s further access to needed health services [3], threaten a household’s ability to pay for other subsistence goods such as food [2, 7–9], and ultimately push them into medical poverty trap [2].

Globally, over 150 million people experienced catastrophic health expenditure annually during the period 1990-2003 [10], while this number increased to 808 million in 2010 [11]. More worryingly, the actual economic burden of health expenditure is less known [2, 4, 6–8, 11–36], due to differences in the measurements of ability to pay (i.e., budget share method - total household income/expenditure or capacity to pay method - actual food spending, partial normative food spending, or normative spending on food, housing, and utilities) [6, 9, 21, 28, 29, 37] and/or in catastrophic thresholds (ranging from 2.5% to 40%) [6, 10, 21, 24, 29]. Although findings on various estimates of catastrophic health expenditure are counterintuitive to researchers, planners, and policymakers [9, 28], they facilitate approaching the issue of horizontal equity (i.e., households in similar circumstances contribute in similar ways to health financing) [30]. However, the distribution of catastrophic health expenditure is not yet well understood [30, 31], which may help us identify the degree to which vertical equity (i.e., the advantaged contribute more to health financing than the disadvantaged) is being addressed or should be handled in the future [30].

Households who have higher out-of-pocket payments on healthcare are, in theory, likely to incur catastrophic health expenditure, regardless of their socioeconomic status (SES) [2, 38]. A 1% increase in the share of out-of-pocket payments to total health expenditure links to a 2.2% increase in the incidence of catastrophic health expenditure [2]. Nevertheless, higher health costs do not always increase the risk for catastrophic health expenditure [2, 10]. Catastrophic health expenditure often disproportionately affects households with lower SES [4, 8, 12–25], as households with a sufficient capacity to buffer their medical expenditure may prevent themselves from catastrophic health expenditure [24], but even a lower level of health spending can make the poor financially disastrous [2, 4, 7, 9, 24, 31, 32]. It is thus necessary to identify the context-specific thresholds of health expenditure that can cause financial catastrophe [31]. According to the vertical equity principle, variable thresholds taking household’s SES into account [24, 31] are more suitable for catastrophe measurement, especially in LMICs with high economic inequalities [4, 31, 32]. Under this circumstance, catastrophic threshold levels increase with household’s SES [32]; therefore, the low-SES can be given more financial protection against financial catastrophe in health than the high-SES [31]. However, variable thresholds have less frequently been used for estimates of catastrophic health expenditure in the literature.

In China, the financial burden of health expenditure is massive [7, 39], due to the rising burden caused by non-communicable diseases (NCDs). Given its chronic nature and greater needs for long-term medical treatment, having NCDs, in theory, increases health service use, thereby posing a major threat to household economics [40]. Two recent cross-country studies suggest that the incidence of catastrophic health expenditure in China was much higher than that in countries with similar levels of economic development [11, 41]. Moreover, the World Health Organization (WHO) reported that, 17.71% of Chinese citizens spent more than 10% of their total household budget on health out-of-pocket payment in 2007 [3], and the incidence of catastrophic health expenditure rose to 23.98% in 2016 [42]. As the population ages, there will be a sharp growth in the prevalence of NCDs in China, causing a more massive economic burden on households [7, 39].

Previous research in China shows marked variation in the incidence of catastrophic health expenditure [7, 8, 12–17, 19, 20, 27, 33–36], the majority of which was conducted at the provincial level [7, 13, 15, 16, 19, 20, 35] or in rural areas [7, 13, 14, 19]. However, evidence from nationally representative survey data is not yet fully available. Moreover, prior Chinese studies have often adopted fixed threshold levels to assess financial catastrophe [7, 8, 12–17, 19, 20, 27, 33–36], but variable thresholds have rarely been used. Furthermore, the relationship between SES and catastrophic health expenditure, in the context of China, is not well understood, where SES was measured variously by health insurance [13, 14, 17, 33, 35], income [19, 35], or education [35]. It is generally accepted that consumption expenditure can accurately measure people’s living standards, particularly in LMICs [12, 23, 31]. Nevertheless, little is known about whether the incidence of catastrophic health expenditure varies across financial capacity in China. The distribution of catastrophic health expenditure across consumption expenditure may help us better understand socioeconomic disparities in catastrophic health expenditure in China. Additionally, prior studies in China often adopted cross-sectional study designs [7, 12, 13, 15, 16, 20, 27, 35, 36], focusing on specific population groups [7, 13, 20, 35] such as cancer patients [20, 35]. It is evident that the determinants of catastrophic health expenditure often change over time [34]; therefore, using longitudinal panel data to examine the impact of financial capacity on catastrophic health expenditure at the national level is warranted.

To address these gaps in the literature, our study aims to use nationally representative data in China to estimate the incidence of catastrophic health expenditure at both fixed and variable thresholds, examine the trend in catastrophic health expenditure over time, and investigate whether catastrophic health expenditure differs across financial capacity at various threshold levels. Systematically monitoring catastrophic health expenditure in China and accurately identifying households at higher risk for catastrophic health expenditure may facilitate the design of national health system [2] and the development of more targeted health financing policies [7], thereby achieving the SDGs by 2030 [3, 5, 6, 8, 11, 37, 41].

## Methods

### Data source and study population

This research used longitudinal data from four waves of the China Health and Retirement Longitudinal Study (CHARLS) conducted in 2011, 2013, 2015, and 2018 (available at: http://charls.pku.edu.cn/en). The CHARLS is a nationally representative household-based longitudinal survey of middle-aged and above adults, conducted by the National School of Development China (Center for Economic Research) at Peking University [43]. Samples in the CHARLS were selected using a four-stage probability-proportional-to-size sampling technique, stratified by per-capita Gross Domestic Product of urban districts and rural counties [43, 44]. A detailed description of the sampling technique of the CHARLS has been reported elsewhere [43].

The CHARLS baseline survey was conducted in 2011-12 [43], with wave 2 in 2013, wave 3 in 2015, and wave 4 in 2018. The baseline survey involves 17,708 individual respondents in 10,257 households, covering 28 of 30 provinces excluding Tibet, 150 countries/districts, 450 villages/urban communities [43, 44]. Of 17,708 individuals, 52.1% were females and 40% were adults aged 60 years and older [43]. In terms of age group and gender distributions, the CHARLS is quite similar to the 2010 Chinese population census [43].

Due to many reasons such as migration or death, a total of 6,953 households were included in all four survey waves and finally included in our study. A balanced panel was adopted in this research, as an observation of the same household in every survey year has the potential to decrease the bias caused by the household heterogeneity.

### Variables

#### Independent variable

SES in our study was measured by annual per-capita household expenditure, which has been widely used to analyze health equity using household survey data [6, 10, 23, 29, 31, 45]. Yearly per-capita household expenditure was calculated using annual total household non-food expenditure (i.e., total household expenditure minus food expenditure) divided by equivalent household size.

In the CHARLS, total household expenditure includes food expenditure, household utility bills (vehicle or home repairs, etc.), purchases of durable goods, education and health expenditures, discretionary spending items (entertainment, etc.), transportation costs, fees (taxes, etc.), and remittances [46]. The missing data on household expenditure were imputed according to participant’s age, rurality of residence, and employment status [47]. Missing data represented no more than 1.2% in 2011 and 1% in 2018 for household expenditure, which are considered as negligible non-response rates. The equivalent household size suggests the number of consumption equivalents in the household [2], calculated as the actual household size to the 0.56 power (i.e., actual household size ^0.56^).

Household’s SES was then evenly divided into quintiles (Q1-Q5) based on annual per-capita household expenditure. Q1 represents the lowest 20% expenditure bracket, whereas Q5 is the top 20% expenditure bracket.

#### Dependent variable

Our study adopted the WHO’s approach to defining catastrophic health expenditure, taking equity concerns into account [2, 10, 28, 38]. Household’s out-of-pocket payments on healthcare are catastrophic when they exceed a specific threshold of their capacity to pay [2, 7–14, 17, 24, 28, 31, 34–36, 38, 44]. Household’s health out-of-pocket payment was defined as all self-paid expenses of outpatient care, inpatient care, and pharmaceuticals [46]. The missing data on out-of-pocket payments were imputed according to the type of NCDs, the type of medical facilities visited, and health insurance status [47]. Missing data represented no more than 0.3% in 2011 and 0.5% in 2013 for out-of-pocket payments, which are considered as negligible non-response rates.

A household’s capacity to pay refers to “effective income remaining after basic subsistence needs have been met” [2]. Total household expenditure is often seen as effective income in the literature, as it can more accurately reflect a household’s purchasing power, compared to total household income [2]. Capacity to pay in our research was defined as household’s non-subsistence expenditure when subsistence spending was less than food expenditure; otherwise, non-food expenditure was used as a proxy for capacity to pay [12, 14, 19, 22, 24, 25, 38]. Subsistence expenditure was calculated as multiplying food-based poverty line by equivalent household size [2, 7, 12, 14, 19, 22, 24, 25, 36, 38]. Given the poorer the household, the higher share of food expenditure to their total consumption [2], poverty line in this study was defined as the average food expenditure of households whose share of food spending to total expenditure was within the 45^th^ and 55^th^ percentiles of the entire study sample [2].

Despite no consensus regarding the specific threshold for defining catastrophic health expenditure [10], threshold level is commonly set at 40% in the literature [2, 7, 8, 12–15, 17, 20, 22, 24, 25, 34–36]. Variable thresholds are more appropriate for the examination of financial catastrophe [4, 31, 32], as the poorer should have greater demands for financial protection than the richer. However, little research has estimated catastrophic health expenditure at variable thresholds. In our study, threshold levels were thus based on a fixed threshold (40%) for all SES groups and a set of variable thresholds for different SES groups. The method used for calculating variable thresholds in our study was applied in previous research in Nigeria [31]. The lowest quintile (Q1) was first selected as “index” quintile, and 40% was selected as the threshold level for Q1. The thresholds for other quintiles were estimated by multiplying 40% by the ratio of mean equivalent expenditure on food for different quintiles [31], as follows:

$$Zi = 40\% \times ({Exp}_{i} / {Exp}_{n})$$

where *Z*_*i*_ = the threshold used for the i^th^ quintile, *Exp*_*i*_ = food expenditure in the i^th^ quintile, and *Exp*_*n*_ = food expenditure in the index quintile.

Two alternative approaches were then indexing the threshold (40%) to the middle quintile (Q3) and the highest quintile (Q5), separately. The threshold levels for remaining quintiles were calculated using an analogous procedure. The threshold level would be adjusted to 100% if it were greater than 100%.

Table 1 shows the threshold levels for catastrophic health expenditure by annual per-capita household expenditure quintile over years.

**Table 1:** Thresholds (%) for catastrophic health expenditure across annual per-capita household expenditure quintile over years.

**Quintiles**^**a**^	**2011**				**2013**				**2015**				**2018**			
	**Fixed threshold**	**Variable thresholds**			**Fixed threshold**	**Variable thresholds**			**Fixed threshold**	**Variable thresholds**			**Fixed threshold**	**Variable thresholds**		
		**Q1 = 40%**^**b**^	**Q3 = 40%**^**c**^	**Q5 = 40%**^**d**^		**Q1 = 40%**^**b**^	**Q3 = 40%**^**c**^	**Q5 = 40%**^**d**^		**Q1 = 40%**^**b**^	**Q3 = 40%**^**c**^	**Q5 = 40%**^**d**^		**Q1 = 40%**^**b**^	**Q3 = 40%**^**c**^	**Q5 = 40%**^**d**^
Q1	40.00	40.00	21.17	4.64	40.00	40.00	20.61	1.65	40.00	40.00	18.55	1.57	40.00	40.00	29.58	11.23
Q2		57.93	30.66	6.73		59.39	30.60	2.46		64.02	29.70	2.51		46.70	34.54	13.11
Q3		75.59	40.00	8.78		77.63	40.00	3.21		86.23	40.00	3.38		54.09	40.00	15.19
Q4		100.00	92.55	20.31		100.00	100.00	9.74		100.00	100.00	11.02		62.19	45.99	17.46
Q5		100.00	100.00	40.00		100.00	100.00	40.00		100.00	100.00	40.00		100.00	100.00	40.00

^a^Quintile 1 is the poorest and Quintile 5 is the wealthiest

^b^Q1 = 40% means that 40% was selected as the threshold level for Q1, and the thresholds for other quintiles were estimated by multiplying 40% by the ratio of average food expenditure in certain quintile to that in Q1

^c^Q3 = 40% means that 40% was selected as the threshold level for Q3, and the thresholds for other quintiles were estimated by multiplying 40% by the ratio of average food expenditure in certain quintile to that in Q3

^d^Q5 = 40% means that 40% was selected as the threshold level for Q5, and the thresholds for other quintiles were estimated by multiplying 40% by the ratio of average food expenditure in certain quintile to that in Q5

Catastrophic health expenditure in our research was regarded as a dummy variable, where the value “1” and “0” indicates households with and without catastrophic health expenditure, respectively.

#### Covariates

Empirical evidence demonstrates that the incidence of catastrophic health expenditure is related to three types of factors - the characteristics of the household head, the features of the household and/or attributes of the family members, and healthcare utilization and policy-related factors [21, 34].

Accordingly, a number of covariates were considered as potential determinants for catastrophic health expenditure in our study, including: (1) demographic characteristics of the household head such as age, sex (male or female), rurality of residence (urban areas, combination zone between urban and rural areas, or rural areas), marital status (living alone (i.e., separated, divorced, widowed, or unmarried), or married or partnered), ethnicity (Han or other ethnic groups), educational level (illiterate, primary school and below, secondary school, or college school and above (including Two-/Three-Year College/Associate degree, Four-Year College/Bachelor’s degree, Master’s degree, and Doctoral degree)), and health insurance status (no insurance, Urban Employee Basic Medical Insurance (UEBMI), Unified Basic Medical Insurance (UBMI), or others); (2) disease status of the household head (no morbidity, single morbidity (i.e., having one NCD), or multimorbidity (i.e., having two or more coexisting NCDs)); (3) the features of the household such as household size; (4) the type of healthcare utilization of the household head (neither outpatient nor inpatient service use, outpatient service use, inpatient service use, or both outpatient and inpatient service use); and (5) survey years (2011, 2013, 2015, or 2018).

Regarding health insurance, UEBMI and UBMI are social basic health insurance schemes in China. UEBMI is exclusively designed for urban employees, while UBMI is designed for urban residents without formal employment and all rural residents, including New Rural Cooperative Medical Scheme, Urban Residents Basic Medical Insurance, and Urban and Rural Resident Medical Insurance. Other insurance schemes include government medical insurance, medical aid, private medical insurance: purchased by work unit, private medical insurance: purchased by individual, urban non-employed person’s health insurance, long-term care insurance, and other medical insurance [46].

Chronic conditions in our study included disabilities (i.e., physical disabilities, vision problem, hearing problem, brain damage/intellectual disability, and speech impediment) and other 14 NCDs diagnosed by a doctor (i.e., hypertension, diabetes, cancer, dyslipidemia, heart disease, stroke, lung disease, stomach and other digestive diseases, liver disease, kidney disease, asthma, arthritis or rheumatism, emotional problems, and memory-associated disease) [46]. Disabilities were also regarded as one type of NCDs in our research, as they frequently occur due to the presence of NCDs, can decrease individual’s quality of life, and may last for their whole life course [48]. The number of chronic conditions for each participant was individually calculated, the range of which was from 0 to 14.

### Statistical analysis

Descriptive analysis was conducted to describe the sample using frequencies (percentages) or means (standard deviations). The sampling frame in the CHARLS includes three levels - individuals, households, and communities. However, the unit of analysis in this study was household, where health out-of-pocket payment and catastrophic health expenditure were estimated at the household level. There were 6,953 households nested within 442 communities.

Households within the same community may have the shared traits based on their residential areas, but there are potentially many differences between communities, including living arrangements and economic conditions [49]; therefore, using simple logistic regression models may violate the assumption of independent error and contribute to methodological bias [49]. A multilevel model can adjust for possible error dependencies within households and communities and correct for bias in the estimates of the coefficients due to clustering [49]. Hence, multilevel mixed-effects logistic regression models were employed to examine the determinants of catastrophic health expenditure at various thresholds to accommodate the nested effects of household- and community-level determinants, adjusted for all covariates. In addition, to identify whether the influence of per-capita household expenditure on catastrophic health expenditure at various thresholds changed over time, four separate models were run, where per-capita household expenditure, survey years, and interactions between per-capita household expenditure and survey years were included. In the logistic regression analysis, results were presented by adjusted odds ratios (OR) and 95% confidence intervals (CI).

Any result with a two-sided *p*-value ≤ 0.05 was considered as statistically significant. All statistical analyses were performed using Stata/SE 16.0 (StataCorp LP, College Station, Texas).

## Results

### Descriptive statistics

Table 2 indicates descriptive statistics for each of four waves of the CHARLS. A total of 6,953 households were included in our study. The mean age of the participants rose from 59 years in 2011 to 66 years in 2018 (Table 2). 45.97% of the respondents were males, while 54.03% were females (Table 2). The overwhelming majority of the household heads were in rural areas (80.33%), living with a spouse or partner (73.02% to 83.53%), having UBMI (81.22% to 84.12%), or with Han ethnicity (92.22%) (Table 2). Over the study period, the percentage of respondents having multimorbidity increased from 41.65% in 2011 to 72.85% in 2018 (Table 2).

**Table 2:** Characteristics of surveyed households in 2011, 2013, 2015, and 2018 (*N* = 6,953)

**Characteristics**	**2011**		**2013**		**2015**		**2018**	
	**Number (Mean)**	**% (SD)**^**a**^	**Number (Mean)**	**% (SD)**^**a**^	**Number (Mean)**	**% (SD)**^**a**^	**Number (Mean)**	**% (SD)**^**a**^
**Demographics**								
Age (years)	59	9.20	61	9.20	63	9.20	66	9.20
Sex								
Male	3,196	45.97	3,196	45.97	3,196	45.97	3,196	45.97
Female	3,757	54.03	3,757	54.03	3,757	54.03	3,757	54.03
Rurality of residence								
Urban areas	932	13.40	932	13.40	932	13.40	932	13.40
Urban-rural areas	436	6.27	436	6.27	436	6.27	436	6.27
Rural areas	5,585	80.33	5,585	80.33	5,585	80.33	5,585	80.33
Marital status								
Living alone^b^	1,145	16.47	1,299	18.68	1,492	21.46	1,876	26.98
Married or partnered	5,808	83.53	5,654	81.32	5,461	78.54	5,077	73.02
Ethnicity								
Han	6,412	92.22	6,412	92.22	6,412	92.22	6,412	92.22
Other ethnic groups	541	7.78	541	7.78	541	7.78	541	7.78
Education								
Illiterate	1,632	23.47	1,632	23.47	1,632	23.47	1,632	23.47
Primary school and below	2,959	42.56	2,959	42.56	2,959	42.56	2,959	42.56
Secondary school	2,249	32.35	2,249	32.35	2,249	32.35	2,249	32.35
College school and above	113	1.63	113	1.63	113	1.63	113	1.63
Health insurance								
No insurance	270	3.88	127	1.83	117	1.68	96	1.38
UEBMI^c^	821	11.81	847	12.18	878	12.63	851	12.24
UBMI^d^	5,647	81.22	5,772	83.01	5,849	84.12	5,683	81.73
Others^e^	215	3.09	207	2.98	109	1.57	323	4.65
**Disease status**								
No morbidity	1,984	28.53	1,817	26.13	1,298	18.67	778	11.19
Single morbidity	2,073	29.81	1,976	28.42	1,719	24.72	1,110	15.96
Multimorbidity	2,896	41.65	3,160	45.45	3,936	56.61	5,065	72.85
**Household size**	3.80	1.80	5.40	1.85	3.30	0.50	2.43	0.66
**Type of healthcare utilization**								
Neither outpatient nor inpatient service use	5,249	75.49	5,255	75.58	4,874	70.10	4,870	70.04
Outpatient service use	1,126	16.19	1,357	19.52	1,088	15.65	790	11.36
Inpatient service use	386	5.55	166	2.39	679	9.77	951	13.68
Both outpatient and inpatient service use	192	2.76	175	2.52	312	4.49	342	4.92

^a^*SD* Standard deviation

^b^Living alone means separated, divorced, widowed, or unmarried

^c^U*EBMI* Urban Employee Basic Medical Insurance

^d^*UBMI* Unified Basic Medical Insurance

^e^ Others = Government medical insurance, Medical aid, Private medical insurance: purchased by work unit, Private medical insurance: purchased by individual, urban non-employed person’s health insurance, Long-term care insurance, and Other medical insurance

### Out-of-pocket payments and incidence of catastrophic health expenditure at various thresholds

Table 3 shows an actual amount of out-of-pocket payments for each quintile of per-capita household expenditure.

**Table 3:** Out-of-pocket payments for each quintile of per-capita household expenditure over years

**Quintiles**^**a**^	**2011**	**2013**	**2015**	**2018**
Q1	601	1,205	1,444	2,128
Q2	1,338	1,883	2,420	2,676
Q3	1,538	2,721	4,411	3,088
Q4	2,116	4,764	6,543	3,671
Q5	3,450	2,572	3,968	4,231

^a^Quintile 1 is the poorest and Quintile 5 is the wealthiest

At a fixed threshold (40%), 10.90%, 9.46%, 13.23%, and 24.75% of households experienced catastrophic health expenditure in 2011, 2013, 2015, and 2018, respectively (Table 4). While when the threshold level for Q1, Q3, and Q5 of per-capita household expenditure was set at 40%, respectively, the overall incidence of catastrophic health expenditure in 2011 was 6.70%, 11.30%, and 27.34%, respectively (Table 4). Similarly, the overall incidence of catastrophic health expenditure was 6.31%, 11.56%, and 36.90% in 2013; 8.95%, 15.16%, and 38.83% in 2015; and 20.67%, 24.42%, and 36.98% in 2018, separately (Table 4).

**Table 4:** Incidence (%) of catastrophic health expenditure by annual per-capita household expenditure quintile at various thresholds over years

**Quintiles**^**a**^	**2011**				**2013**				**2015**				**2018**			
	**Fixed threshold (40%)**	**Variable thresholds**			**Fixed threshold (40%)**	**Variable thresholds**			**Fixed threshold (40%)**	**Variable thresholds**			**Fixed threshold (40%)**	**Variable thresholds**		
		**Q1 = 40%**^**b**^	**Q3 = 40%**^**c**^	**Q5 = 40%**^**d**^		**Q1 = 40%**^**b**^	**Q3 = 40%**^**c**^	**Q5 = 40%**^**d**^		**Q1 = 40%**^**b**^	**Q3 = 40%**^**c**^	**Q5 = 40%**^**d**^		**Q1 = 40%**^**b**^	**Q3 = 40%**^**c**^	**Q5 = 40%**^**d**^
Q1	15.25	15.25	23.60	41.58	18.56	18.56	29.57	59.50	24.10	24.10	34.68	59.86	46.95	46.95	50.97	61.08
Q2	14.02	9.13	17.47	39.90	12.01	7.33	15.96	55.79	18.33	11.79	22.65	59.17	31.68	28.38	34.41	49.14
Q3	11.36	5.10	11.36	32.85	10.42	3.81	10.42	49.39	15.96	6.33	15.96	55.21	21.05	16.01	21.05	39.47
Q4	8.99	2.95	3.02	17.47	6.04	1.87	1.87	19.55	7.19	2.37	2.37	19.34	16.10	9.47	13.18	27.35
Q5	4.89	1.08	1.08	4.89	0.29	0.00	0.00	0.29	0.58	0.14	0.14	0.58	7.96	2.51	2.51	7.96
**Total**	**10.90**	**6.70**	**11.30**	**27.34**	**9.46**	**6.31**	**11.56**	**36.90**	**13.23**	**8.95**	**15.16**	**38.83**	**24.75**	**20.67**	**24.42**	**36.98**

^a^Quintile 1 is the poorest and Quintile 5 is the wealthiest

^b^Q1 = 40% means that 40% was selected as the threshold level for Q1, and the thresholds for other quintiles were estimated by multiplying 40% by the ratio of average food expenditure in certain quintile to that in Q1

^c^Q3 = 40% means that 40% was selected as the threshold level for Q3, and the thresholds for other quintiles were estimated by multiplying 40% by the ratio of average food expenditure in certain quintile to that in Q3

^d^Q5 = 40% means that 40% was selected as the threshold level for Q5, and the thresholds for other quintiles were estimated by multiplying 40% by the ratio of average food expenditure in certain quintile to that in Q5

Overall, we observed a decreasing trend in the incidence of catastrophic health expenditure from the poorest to the richest quintile at both fixed and variable thresholds (Table 4). We also found that, across each per-capita household expenditure quintile, the incidence of catastrophic health expenditure generally reduced from 2011 to 2013 and then increased between 2013 and 2018, regardless of the threshold levels set (Table 4).

### Multilevel logistic regression models for catastrophic health expenditure at various thresholds

Multilevel logistic regression analysis identified several statistically significant determinants of catastrophic health expenditure at both fixed and variable threshold levels (Table 5).

**Table 5:** Factors associated with the incidence of catastrophic health expenditure at various thresholds^a^

**Characteristics**	**Fixed threshold (40%)**		**Variable thresholds**					
			**Q1 = 40%**^**b**^		**Q3 = 40%**^**c**^		**Q5 = 40%**^**d**^	
	**OR (95% CI)**	***P*-value**	**OR (95% CI)**	***P*-value**	**OR (95% CI)**	***P*-value**	**OR (95% CI)**	***P*-value**
**Survey years**								
2011								
2013	0.975 (0.851-1.118)	0.720	1.049 (0.889-1.237)	0.574	1.119 (0.979-1.280)	0.099	**2.039 (1.844-2.254)**	**<0.0001**
2015	0.971 (0.864-1.092)	0.622	1.093 (0.948-1.260)	0.220	**1.141 (1.014-1.283)**	**0.028**	**1.670 (1.524-1.830)**	**<0.0001**
2018	**2.121 (1.881-2.391)**	**<0.0001**	**3.203 (2.776-3.695)**	**<0.0001**	**2.229 (1.969-2.523)**	**<0.0001**	**1.323 (1.196-1.464)**	**<0.0001**
**Per-capita household expenditure quintiles**^**e**^								
Q5								
Q4	**3.284 (2.728-3.954)**	**<0.0001**	**4.914 (3.586-6.732)**	**<0.0001**	**6.014 (4.416-8.191)**	**<0.0001**	**9.159 (7.706-10.886)**	**<0.0001**
Q3	**6.129 (5.115-7.344)**	**<0.0001**	**10.834 (7.993-14.684)**	**<0.0001**	**24.088 (17.907-32.402)**	**<0.0001**	**37.946 (31.878-45.170)**	**<0.0001**
Q2	**9.284 (7.748-11.125)**	**<0.0001**	**24.607 (18.219-33.236)**	**<0.0001**	**47.824 (35.537-64.358)**	**<0.0001**	**57.029 (47.739-68.127)**	**<0.0001**
Q1	**18.203 (15.131-21.900)**	**<0.0001**	**73.990 (54.523-100.408)**	**<0.0001**	**119.603 (88.411-161.798)**	**<0.0001**	**86.717 (72.179-104.182)**	**<0.0001**
**Education**								
Illiterate								
Primary school and below	**1.158 (1.028-1.304)**	**0.016**	**1.141 (1.002-1.300)**	**0.047**	1.123 (0.998-1.264)	0.053	**1.125 (1.014-1.247)**	**0.026**
Secondary school	**1.173 (1.015-1.357)**	**0.031**	1.162 (0.989-1.365)	0.068	1.127 (0.975-1.303)	0.106	1.111 (0.980-1.259)	0.099
College school and above	0.948 (0.597-1.505)	0.820	0.927 (0.502-1.711)	0.808	1.015 (0.607-1.697)	0.954	1.326 (0.920-1.911)	0.130
**Health insurance**								
No insurance								
UEBMI^f^	1.332 (0.922-1.923)	0.127	1.178 (0.776-1.790)	0.442	1.381 (0.954-1.999)	0.088	**1.520 (1.133-2.038)**	**0.005**
UBMI^g^	1.291 (0.964-1.728)	0.086	1.221 (0.888-1.679)	0.218	**1.437 (1.082-1.909)**	**0.012**	**1.505 (1.200-1.888)**	**<0.0001**
Others^h^	1.139 (0.780-1.664)	0.501	1.098 (0.716-1.684)	0.667	1.213 (0.827-1.779)	0.323	**1.385 (1.022-1.876)**	**0.036**
**Disease status**								
No morbidity								
Single morbidity	**1.432 (1.238-1.657)**	**<0.0001**	**1.380 (1.163-1.636)**	**<0.0001**	**1.598 (1.383-1.846)**	**<0.0001**	**1.415 (1.272-1.574)**	**<0.0001**
Multimorbidity	**2.191 (1.918-2.503)**	**<0.0001**	**2.150 (1.844-2.507)**	**<0.0001**	**2.446 (2.142-2.794)**	**<0.0001**	**2.382 (2.153-2.634)**	**<0.0001**
**Type of healthcare utilization**								
Neither outpatient nor inpatient service use								
Outpatient service use	**3.141 (2.830-3.485)**	**<0.0001**	**3.042 (2.695-3.435)**	**<0.0001**	**3.112 (2.797-3.463)**	**<0.0001**	**2.656 (2.421-2.913)**	**<0.0001**
Inpatient service use	**2.521 (2.208-2.879)**	**<0.0001**	**2.511 (2.156-2.926)**	**<0.0001**	**2.520 (2.191-2.899)**	**<0.0001**	**1.999 (1.766-2.264)**	**<0.0001**
Both outpatient and inpatient service use	**5.508 (4.626-6.558)**	**<0.0001**	**5.746 (4.710-7.010)**	**<0.0001**	**5.562 (4.608-6.714)**	**<0.0001**	**4.225 (3.529-5.058)**	**<0.0001**
**Age (years)**	**1.017 (1.011-1.022)**	**<0.0001**	**1.015 (1.008-1.021)**	**<0.0001**	**1.016 (1.010-1.021)**	**<0.0001**	**1.012 (1.007-1.017)**	**<0.0001**
**Sex**								
Male								
Female	1.098 (0.998-1.208)	0.054	**1.133 (1.018-1.262)**	**0.022**	**1.115 (1.013-1.227)**	**0.027**	**1.089 (1.003-1.181)**	**0.042**
**Rurality of residence**								
Urban areas								
Urban-rural areas	1.059 (0.831-1.349)	0.644	1.181 (0.886-1.574)	0.257	1.144 (0.888-1.476)	0.298	0.919 (0.748-1.128)	0.418
Rural areas	0.980 (0.780-1.232)	0.865	1.015 (0.775-1.330)	0.912	0.983 (0.775-1.248)	0.890	0.931 (0.767-1.129)	0.466
**Marital status**								
Living alone^i^								
Married or partnered	**2.717 (2.405-3.069)**	**<0.0001**	**2.504 (2.192-2.861)**	**<0.0001**	**2.787 (2.470-3.145)**	**<0.0001**	**3.026 (2.734-3.350)**	**<0.0001**
**Ethnicity**								
Han								
Other ethnic groups	**1.237 (1.025-1.491)**	**0.026**	1.168 (0.952-1.435)	0.137	1.165 (0.961-1.412)	0.121	1.107 (0.928-1.321)	0.258
**Household size**	**0.840 (0.809-0.872)**	**<0.0001**	**0.848 (0.811-0.887)**	**<0.0001**	**0.871 (0.840-0.903)**	**<0.0001**	**0.907 (0.883-0.932)**	**<0.0001**
**Variance components**	**Variance**	**Standard deviation**	**Variance**	**Standard deviation**	**Variance**	**Standard deviation**	**Variance**	**Standard deviation**
Household	0.540	0.066	0.459	0.081	0.490	0.066	0.625	0.052
Community	0.181	0.028	0.137	0.028	0.192	0.029	0.264	0.030
**Akaike Information Criterion (AIC)**	18934.92		14292.06		18135.98		27199.96	
**Bayesian Information Criterion (BIC)**	19165.45		14522.59		18366.51		27430.49	

^a^Odds ratios (OR), 95% confidence intervals (CI), and *p*-value significantly related to catastrophic health expenditure are bolded

^b^Q1 = 40% means that 40% was selected as the threshold level for Q1, and the thresholds for other quintiles were estimated by multiplying 40% by the ratio of average food expenditure in certain quintile to that in Q1

^c^Q3 = 40% means that 40% was selected as the threshold level for Q3, and the thresholds for other quintiles were estimated by multiplying 40% by the ratio of average food expenditure in certain quintile to that in Q3

^d^Q5 = 40% means that 40% was selected as the threshold level for Q5, and the thresholds for other quintiles were estimated by multiplying 40% by the ratio of average food expenditure in certain quintile to that in Q5

^e^Quintile 1 is the poorest and Quintile 5 is the wealthiest

^f^*UEBMI* Urban Employee Basic Medical Insurance

^g^*UBMI* Unified Basic Medical Insurance

^h^Others = Government medical insurance, Medical aid, Private medical insurance: purchased by work unit, Private medical insurance: purchased by individual, urban non-employed person’s health insurance, Long-term care insurance, and Other medical insurance

^i^Living alone means separated, divorced, widowed, or unmarried

The likelihood of suffering catastrophic health expenditure in 2018 (fixed: OR = 2.121; Q1 = 40%: 3.203; Q3 = 40%: 2.229; Q5 = 40%: 1.323) was higher than that in 2011 (Table 5). Similarly, compared to households in the highest quintile of per-capita household expenditure (Q5), those in Q4 (fixed: OR = 3.284; Q1 = 40%: 4.914; Q3 = 40%: 6.014; Q5 = 40%: 9.159), in Q3 (fixed: 6.129; Q1 = 40%: 10.834; Q3 = 40%: 24.088; Q5 = 40%: 37.946), in Q2 (fixed: 9.284; Q1 = 40%: 24.607; Q3 = 40%: 47.824; Q5 = 40%: 57.029), or in Q1 (fixed: 18.203; Q1 = 40%: 73.990; Q3 = 40%: 119.603; Q5 = 40%: 86.717) were more likely to suffer catastrophic health expenditure (Table 5). Moreover, compared to households in Q5, the odds of suffering catastrophic health expenditure in those in lower quintiles were generally inflated over time, regardless of the thresholds set, suggesting that the effect of per-capita household expenditure on the incidence of catastrophic health expenditure significantly changed over time (Supplementary Table 1).

Furthermore, compared to households headed by an individual with no morbidity, those having single morbidity were more likely to experience catastrophic health expenditure at both fixed (OR = 1.432) and variable (Q1 = 40%: 1.380; Q3 = 40%: 1.598; Q5 = 40%: 1.415) thresholds. Multimorbidity (fixed: OR = 2.191; Q1 = 40%: 2.150; Q3 = 40%: 2.446; Q5 = 40%: 2.382) was also related to greater risks for catastrophic health expenditure, irrespective of the thresholds set (Table 5). Likewise, having health service use (including outpatient, inpatient, and both outpatient and inpatient) increased the likelihood of catastrophic health expenditure, no matter how the threshold levels were set (Table 5).

Higher risks for catastrophic health expenditure were also found for increased age and being married or partnered, regardless of the thresholds set (Table 5). In contrast, household size was negatively associated with the odds of catastrophic health expenditure at both fixed (OR = 0.840) and variable (Q1 = 40%: 0.848; Q3 = 40%: 0.871; Q5 = 40%: 0.907) thresholds (Table 5).

## Discussion

Using four waves of the CHARLS data, this study revealed that the incidence of catastrophic health expenditure varied, depending on the thresholds set. At a fixed threshold (40%), 10.90%, 9.46%, 13.23%, or 24.75% of households suffered catastrophic health expenditure in 2011, 2013, 2015, and 2018, respectively, which were generally much lower than those at variable thresholds. This study also found that the likelihood of incurring catastrophic health expenditure generally decreased from 2011 to 2013, and then there was a rising trend between 2013 and 2018. Additionally, lower per-capita household expenditure quintiles, diagnosed with NCDs, and having healthcare utilization increased the odds of catastrophic health expenditure, irrespective of the thresholds set. To the best of our knowledge, our findings suggest a national picture on the incidence of catastrophic health expenditure at various thresholds in China and changes in its incidence over years and provide empirical evidence on the effect of financial capacity on catastrophic health expenditure.

### Incidence of catastrophic health expenditure

The incidence of catastrophic health expenditure often differs substantially in the literature [2, 4, 6–8, 11–36, 50], due to its various measurements [6, 9, 10, 28, 37]. As budget share method (health out-of-pocket payment exceeding 10% or 25% threshold of total household income/expenditure) is often criticized for underestimating the economic burden of health expenditure on the poor [4, 6, 10, 28, 30], the WHO’s approach (i.e., capacity to pay method) is often recommended to assess financial catastrophe [2, 10, 28, 38]. Despite as a better proxy for a household’s ability to pay [2, 10, 28, 38], the WHO’s approach is unable to reflect how far out-of-pocket payments on health consume a household’s resources that are required for non-medical necessities such as food [37]. Consequently, both budget share method and capacity to pay method have limitations in catastrophe measurement. Another concern is no consensus regarding the specific catastrophic threshold for financial catastrophe [10], thereby making cross-country comparisons difficult [9, 30]. Uniform thresholds have frequently been used, but fail to capture the experience of households with low SES [2, 4, 31, 32]. To overcome this limitation, variable thresholds were used in our research, taking vertical equity concerns into consideration. This study suggesteds that, when the threshold for Q1, Q3, and Q5 of per-capita household expenditure was set at 40%, separately, the overall incidence of catastrophic health expenditure was 6.70%, 11.30%, and 27.34% in 2011; 6.31%, 11.56%, and 36.90% in 2013; 8.95%, 15.16%, and 38.83% in 2015; and 20.67%, 24.42%, and 36.98% in 2018, respectively. Likewise, previous studies using variable thresholds indicate that the incidence of catastrophic health expenditure was 32.00% (Q1 = 5%) or 36.50% (Q5 = 40%) in Nigeria [31], whereas 13.00% of households incurred catastrophic health expenditure in South Africa [4].

To examine the exact financial burden of health spending, comparisons have often been conducted in prior studies, mainly from two perspectives - using different methods for catastrophic health expenditure [3, 4, 6, 23, 28, 30, 32, 33] or using the same method but at various thresholds [4, 6, 19, 24, 30–32]. According to different approaches to defining catastrophic health expenditure, most research shows that its incidence differed [3, 4, 23, 28, 30, 32, 33]. A prior study in China reported that the incidence of catastrophic health expenditure increased from 20.86% in 2011 to 31.00% in 2015 at the 40% threshold of non-food expenditure, while it rose from 29.92% in 2011 to 39.42% in 2015 at the 10% threshold of total consumption expenditure [33]. Similarly, depending on its definition, the incidence of catastrophic health expenditure ranged from 0.4% to 2.1% in Liberia [30]. Furthermore, catastrophic health expenditure was also compared using the same approach but at various thresholds [4, 6, 19, 24, 30–32]. Evidence from rural China suggests that catastrophic health expenditure reduced from 13.62% in 2009 to 7.74% in 2010 at the 40% threshold of a household’s capacity to pay, while it decreased from 16.85% to 11.75% (30% threshold) or from 10.60% to 5.51% (50% threshold) [19]. Likewise, according to budget share method, a cross-country analysis involving 14 European countries found that catastrophic health expenditure ranged from over 2% in Czechia to around 33% in Georgia at the threshold of 10%, while it varied from 0% in Czechia to 9% in Georgia at the threshold of 25% [6]. Nevertheless, few studies have compared catastrophic health expenditure at fixed thresholds with that at variable thresholds [4, 31, 32]. This study adopted non-subsistence/non-food expenditure as a proxy for a household’s capacity to pay and found great differences in catastrophic health expenditure at various thresholds, which consolidates prior findings [4, 6, 19, 30–32]. This research also revealed that the incidence of catastrophic health expenditure is generally higher at variable thresholds than that at a fixed threshold, which is consistent with empirical evidence that using variable thresholds contributes to higher overall and disaggregated levels of catastrophe [31].

Although this study adopted both fixed and variable thresholds to measure its incidence, catastrophic health expenditure is subject to its inability to fully capture how health needs affect household resources [10, 30, 37]. Catastrophic health expenditure only measures the financial consequences of paying for health services [10], which generally ignores households who are unable to access or afford health care but suffer financial distress [10, 11, 21, 30, 31, 40, 41]. Due to the underestimation of households without financial protection in health [10, 28, 30], some misleading findings occur [30]. For example, the WHO reported that households from low-income countries were more financially protected than those from middle- and high-income countries [3]. Moreover, the standard definition of catastrophic health expenditure often neglects the role of alternative coping strategies (e.g., savings, borrowings, or mortgaging or selling assets) in health financing [4, 21, 27, 28, 40] and the life-time consequences of health shocks [28, 37]. Hence, to design more targeted health policies, further developing how to accurately measure financial risk protection is needed [11, 30, 37, 41], particularly in countries with high economic inequalities [28]. There is evidence that estimating healthcare forgone can somewhat complement the limitation in the standard catastrophic health expenditure measurements and should thus be included in the future analysis of financial risk protection [30].

### Distribution of catastrophic health expenditure

It is generally accepted that catastrophic health expenditure disproportionately affects the lower-SES [4, 8, 12–25], where SES was measured by wealth [4], educational level [12, 35], health insurance [12–18, 23, 35, 45], income [8, 19–21, 25, 35], poverty [22], consumption expenditure [23, 33, 45], or employment [12, 18, 24]. Consistent with these results, this study found that lower per-capita household expenditure quintiles were related to higher risks for catastrophic health expenditure, no matter how the threshold levels were set. This is possibly because fee-for-service is the major provider payment method in China [8, 12, 35, 51], mainly including deductibles (i.e., out-of-pocket payments below deductible thresholds), co-payments (i.e., a certain percentage applied to the fees above deductibles but below the reimbursement ceiling), and patient payments beyond the reimbursement ceiling (i.e., out-of-pocket payments over the upper limit of co-payments) [51]. Given reducing government subsidies [35], health providers in China have strong incentives to maximize their revenue through increasing health service volume [12, 35]. Therefore, over-treatment and over-prescription are not uncommon in China [52]. To minimize unnecessary health service use, higher deductibles and co-insurance payments have been subsequently introduced [53]; thus, the low-SES are often unable to afford needed health services [54]. To meet their health needs, households with low SES may have to decrease other subsistence spending [9], the abandonment of which will, in turn, lead to higher economic risks [9], higher likelihood of poverty [9], and lower quality of life [9, 21, 27].

In addition to risk-sharing across SES, the fairness in health financial contribution also includes risk-pooling between the healthy and the sick [38]. This research suggested that single morbidity and multimorbidity increased the odds of catastrophic health expenditure, regardless of the threshold levels set, which is consistent with previous findings [12, 21, 23, 24, 40, 44, 52]. The possible explanation could be that benefits packages and reimbursement ratios vary significantly across health insurance schemes in China, especially for patients with NCDs [8, 55]. Given fee-for-service and single disease-based payment system [39], reforming cost- and risk-sharing arrangements is warranted in China [12]. Introducing a comprehensive payment system may play a role, where coexisting NCDs can be treated and reimbursed efficiently [39]. In addition, it is necessary to further explore which combinations of NCDs are the major contributor to catastrophic health expenditure, as different combinations of chronic conditions often require different preventive care and medical treatment [39].

This research also showed that advanced age and having health service use were associated with greater risks for catastrophic health expenditure at various thresholds. Similarly, prior research reported that older age [12] and healthcare utilization (e.g., outpatient [15, 33], inpatient [33, 34], or both outpatient and inpatient [25] care) were positively related to the incidence of catastrophic health expenditure. Our findings that health service use increased the odds of catastrophic health expenditure indicate that the financial support provided by health insurance schemes may be offset by increased healthcare utilization and service charges [8, 12], and consequently has a limited effect on financial risk protection [8, 12]. More worryingly, the increasing flow of government subsidies to health insurance companies would further increase health service volumes and charges through provider-induced demand, if no effective cost-control measures were implemented [12].

### Catastrophic health expenditure issue in China and future efforts

This research suggested that the incidence of catastrophic health expenditure in China is generally higher than that reported in other LMICs [4, 11, 25, 28–32, 41, 50], irrespective of how catastrophic health expenditure is defined. The potential explanations could be fee-for-service payment mechanism [8, 12, 35, 51]. High dependence on out-of-pocket payments is a critical concern [51], particularly for NCDs due to its chronic nature [40]. Moving from fee-for-service payment method to prepaid coverage may play a significant role in strengthening financial protection [54]. Evidence from a multi-country analysis shows that catastrophic health expenditure was negatively associated with the share of total health expenditure channeled through social security funds or other government financial protection arrangements [11]. Hence, further raising the percentage of total health spending that is prepaid, especially through taxes and mandatory contributions, is necessary [11]. Another potential reason could be inadequate financial protection from benefit packages of health insurance schemes [8, 17, 51]. Great strides in the fraction of the Chinese population covered by health insurance may, to some extent, mitigate the financial distress due to illness [3, 7, 8, 12, 56]. However, increased insurance coverage failed to effectively decrease the incidence of catastrophic health expenditure [11, 17], largely due to significant differences in basic health insurance schemes, in terms of target population, financing mechanism, compensation level, and reimbursement mechanism [34, 35, 51]. Even for the same health insurance plan, benefit packages also differ by city and province [34, 51], owing to various fiscal capacity of local governments [51]. Therefore, the current mix of social health insurance schemes in China may hinder equal access to healthcare and have a limited influence on financial protection [12, 35].

This research also found a declining trend in the incidence of catastrophic health expenditure from 2011 to 2013 at a fixed threshold and at most variable thresholds, which is consistent with prior findings in China [8, 13, 14, 19, 34]. For example, among Chinese adults aged over 16 years, the incidence of catastrophic health expenditure was reported to drop from 19.37% in 2010 to 15.11% in 2016 [8] or from 14.70% in 2010 to 8.70% in 2018 [34]. Since the World Health Assembly in 2005, China has committed to improving financial risk protection in health [4], aiming to ensure that all residents can enjoy affordable universal basic healthcare [8, 33, 54]. However, due to the rising expansion of health insurance schemes, people’s demands for medical services have dramatically grown, particularly among the elderly, which leads to higher risk for catastrophic health expenditure [33]. The mean age of our study population was as high as 66 years in 2018; thus, it seems not surprising to observe a growing trend in catastrophic health expenditure from 2013 to 2018, which consolidates prior findings in China [17, 33]. Furthermore, despite high insurance coverage in China, patient cost-sharing remains extremely high for both outpatient and inpatient care services, and medication fees often account for around half of total health spending [50]. Given patient cost-sharing as an important indicator of financial protection against illness and equity in health financing [54], decreasing patient cost-sharing may play a major role in attenuating the massive economic burden on households in China [51].

Given the increasingly undue financial burden facing households in China [11, 28, 30, 31, 41, 50], it is imperative to expand funding pools [7, 10], redesign provider payment method [12, 39], and redevelop social health insurance schemes [8, 12, 57]. The financial burden is particularly born by the socioeconomically disadvantaged in China; hence, particular attention should be given to the low-SES. Further expanding social health insurance schemes (e.g., critical illness insurance) and medical financial assistance program is needed [8, 12, 56]. It is also worth noting that the benefits of increased health insurance coverage appear to be offset by the rising medical costs and health demands [58]. Since the 1980s, there has been a sharp increase in health costs in China, due to constant changes in health financing policies (e.g., fee-for-service payment mechanism) [54]. It is thus necessary to roll out more coordinated supply-side reforms targeting cost containment [8, 12, 59], as constraint-oriented health policies, especially in the domain of health service delivery, may effectively mitigate the undue financial burden [59].

### Limitations, strengths and policy implications

This study is subject to some disadvantages; therefore, results should be interpreted with caution. First, the recall period for outpatient and pharmaceutical services was monthly, while the recall period for inpatient services was yearly [46]. Moreover, health expenditure was self-reported and thus subject to recall bias [51]. Health costs were only calculated for those generated from formal health sectors, whereas those from informal care sectors were unknown [46]. Second, despite catastrophic health expenditure defined at various thresholds in our research, the exact economic burden on Chinese households is not fully measured. This is potentially because the measurements of catastrophic health expenditure fail to consider the socioeconomically disadvantaged who forgo needed healthcare when health services are inaccessible or unaffordable [10, 11, 21, 30, 31, 40, 41]. Comprehensively evaluating financial risk protection due to illness is thus warranted [11, 30, 37, 41]. Third, owing to the data availability, only household heads aged over 45 years were included in this study, potentially resulting in biased estimations of influencing determinants of catastrophic health expenditure [35]. Lastly, the characteristics of the household head were considered as the covariates in this study, which may lead to an underestimation of the results (adjusted estimates of the effect of SES on catastrophic health expenditure). However, the characteristics of the household head tend to better represent the overall household characteristics [8, 12, 19, 34].

Despite these limitations, our findings showed that the incidence of catastrophic health expenditure in China varied at various thresholds and was much higher at variable thresholds. Our findings also indicated a decreasing trend in the incidence of catastrophic health expenditure from 2011 to 2013 and then a rising trend afterwards. Additionally, our findings provided new evidence on inequalities in catastrophic health expenditure by household’s financial capacity. These findings may inform the development of more targeted policies and interventions to address the growing economic burden of health spending, particularly for the socioeconomically disadvantaged and those with NCDs, and ultimately to advance the achievement of the SDGs by 2030. Results from our study may also shed light on health system design and future health reform in China and can be applicable to other LMICs.

## Conclusions

Using the nationally representative CHARLS data in 2011-2018, this study found that depending on the threshold levels set, the incidence of catastrophic health expenditure differed, and such incidence was generally higher at variable thresholds than that at a fixed threshold. There was generally a reducing trend in catastrophic health expenditure from 2011 to 2013 and then a rising trend afterwards. Lower per-capita household expenditure quintiles, diagnosed with NCDs, and having health service use increased the risks for catastrophic health expenditure, regardless of the thresholds set. The financial protection against catastrophic health expenditure remains a challenge in China, particularly for the socioeconomically disadvantaged and those with NCDs. Hence, UHC financial protection should be further strengthened in China to effectively prevent households from economic losses in case of illness.

### Supplementary Information

**Additional file 1:** **Supplementary Table 1.** Interaction effects between per-capita household expenditure quintiles and survey years for catastrophic health expenditure at various thresholds^a^.

## Data Availability

The datasets generated and/or analyzed during the current study are publicly available in the CHARLS repository, http://charls.pku.edu.cn/en.

## Abbreviations

CHARLS: China Health and Retirement Longitudinal Study
CI: Confidence intervals
LMICs: Low- and middle-income countries
NCDs: Non-communicable diseases
OR: Odds ratios
SDGs: Sustainable Development Goals; SES: Socioeconomic status
UBMI: Unified Basic Medical Insurance
UEBMI: Urban Employee Basic Medical Insurance
UHC: Universal health coverage
WHO: World Health Organization
