# Supplementary material for: Catastrophic health expenditure and its association with socioeconomic status in China: evidence from the 2011-2018 China Health and Retirement Longitudinal Study
Source: Int J Equity Health. 2023 Sep 21;22:194. doi: 10.1186/s12939-023-02008-z (PMC10515247; doi:10.1186/s12939-023-02008-z)
Supplement: Supplementary file 1 — Additional file 1: Supplementary Table 1. Interaction effects between per-capita household expenditure quintiles and survey years for catastrophic health expenditure at various thresholdsa. [file 12939_2023_2008_MOESM1_ESM.docx]

**Supplementary Table 1.** Interaction effects between per-capita household expenditure quintiles and survey years for catastrophic health expenditure at various thresholds.^a^

| **Characteristics** | **Fixed threshold (40%)** | |  | | **Variable thresholds** | | | | | | | |
| --- | --- | --- | --- | --- | --- | --- | --- | --- | --- | --- | --- | --- |
|  |  |  |  | | **Q1 = 40%^b^** | |  | **Q3 = 40%^c^** | |  | **Q5 = 40%^d^** | |
|  | **OR (95% CI)** | ***P*-value** |  | **OR (95% CI)** | | ***P*-value** |  | **OR (95% CI)** | ***P*-value** |  | **OR (95% CI)** | ***P*-value** |
| **Per-capita household expenditure quintiles^e^** | | | | | | | | | | | | |
| Q5 | | | | | | | | | | | | |
| Q4 | **1.957 (1.410-2.716)** | **<0.0001** |  | **2.718 (1.479-4.993)** | | **0.001** |  | **2.799 (1.523-5.143)** | **0.001** |  | **4.902 (3.608-6.661)** | **<0.0001** |
| Q3 | **2.766 (2.013-3.802)** | **<0.0001** |  | **5.144 (2.896-9.137)** | | **<0.0001** |  | **13.585 (7.839-23.541)** | **<0.0001** |  | **14.173 (10.544-19.051)** | **<0.0001** |
| Q2 | **3.618 (2.651-4.936)** | **<0.0001** |  | **9.917 (5.702-17.248)** | | **<0.0001** |  | **23.750 (13.807-40.853)** | **<0.0001** |  | **20.703 (15.412-27.810)** | **<0.0001** |
| Q1 | **4.548 (3.338-6.198)** | **<0.0001** |  | **20.832 (12.094-35.882)** | | **<0.0001** |  | **41.881 (24.397-71.894)** | **<0.0001** |  | **27.333 (20.301-36.801)** | **<0.0001** |
| **Survey years** | | | | | | | | | | | | |
| 2011 | | | | | | | | | | | | |
| 2013 | **0.059 (0.021-0.163)** | **<0.0001** |  | 0.628 (0.378-1.046) | | 0.074 |  | 0.614 (0.369-1.022) | 0.061 |  | **0.060 (0.021-0.165)** | **<0.0001** |
| 2015 | **0.120 (0.057-0.254)** | **<0.0001** |  | **0.140 (0.032-0.617)** | | **0.009** |  | **0.145 (0.033-0.639)** | **0.011** |  | **0.126 (0.060-0.267)** | **<0.0001** |
| 2018 | **1.777 (1.276-2.476)** | **0.001** |  | **2.402 (1.292-4.464)** | | **0.006** |  | **2.452 (1.317-4.568)** | **0.005** |  | **1.823 (1.306-2.545)** | **<0.0001** |
| **Per-capita household expenditure quintiles^e^ * Survey years** | | | | | | | | | | | | |
| Q5^e^ * 2011 | | | | | | | | | | | | |
| Q4 * 2013 | **10.738 (3.697-31.184)** | **<0.0001** |  | Empty | | |  | Empty | |  | **20.445 (7.187-58.160)** | **<0.0001** |
| Q4 * 2015 | **6.622 (2.962-14.805)** | **<0.0001** |  | **5.899 (1.242-28.013)** | | **0.026** |  | **5.614 (1.181-26.689)** | **0.030** |  | **9.388 (4.294-20.526)** | **<0.0001** |
| Q4 * 2018 | 1.255 (0.824-1.913) | 0.291 |  | 1.605 (0.777-3.318) | | 0.201 |  | **2.381 (1.157-4.900)** | **0.018** |  | 1.142 (0.768-1.698) | 0.512 |
| Q3 * 2013 | **14.838 (5.176-42.540)** | **<0.0001** |  | 1.133 (0.599-2.142) | | 0.701 |  | 1.427 (0.804-2.535) | 0.225 |  | **39.088 (13.846-110.350)** | **<0.0001** |
| Q3 * 2015 | **12.388 (5.641-27.203)** | **<0.0001** |  | **8.799 (1.922-40.276)** | | **0.005** |  | **10.342 (2.300-46.506)** | **0.002** |  | **24.159 (11.161-52.297)** | **<0.0001** |
| Q3 * 2018 | 1.334 (0.887-2.007) | 0.166 |  | 1.690 (0.848-3.367) | | 0.136 |  | 0.962 (0.494-1.873) | 0.909 |  | 0.813 (0.554-1.193) | 0.290 |
| Q2 * 2013 | **13.837 (4.848-39.494)** | **<0.0001** |  | 1.234 (0.686-2.221) | | 0.483 |  | 1.432 (0.820-2.501) | 0.206 |  | **37.904 (13.436-106.926)** | **<0.0001** |
| Q2 * 2015 | **11.612 (5.316-25.366)** | **<0.0001** |  | **9.610 (2.132-43.321)** | | **0.003** |  | **9.859 (2.203-44.115)** | **0.003** |  | **20.980 (9.701-45.373)** | **<0.0001** |
| Q2 * 2018 | **1.902 (1.280-2.826)** | **0.001** |  | **1.979 (1.018-3.850)** | | **0.044** |  | 1.195 (0.621-2.302) | 0.593 |  | 0.886 (0.606-1.295) | 0.531 |
| Q1^e^ * 2013 | **20.405 (7.188-57.925)** | **<0.0001** |  | **1.935 (1.110-3.371)** | | **0.020** |  | **2.135 (1.238-3.682)** | **0.006** |  | **36.433 (12.922-102.719)** | **<0.0001** |
| Q1^e^ * 2015 | **15.025 (6.903-32.704)** | **<0.0001** |  | **12.994 (2.906-58.100)** | | **0.001** |  | **12.179 (2.730-54.340)** | **0.001** |  | **17.513 (8.102-37.858)** | **<0.0001** |
| Q1^e^ * 2018 | **3.536 (2.390-5.231)** | **<0.0001** |  | **2.559 (1.331-4.921)** | | **0.005** |  | 1.643 (0.857-3.149) | 0.135 |  | 1.343 (0.919-1.964) | 0.128 |

Note. ^a^ Odds ratios (OR), 95% confidence intervals (CI), and *p*-value significantly related to catastrophic health expenditure are bolded; ^b^ Q1 = 40% means that 40% was selected as the threshold level for Q1, and the thresholds for other quintiles were estimated by multiplying 40% by the ratio of average food expenditure in certain quintile to that in Q1; ^c^ Q3 = 40% means that 40% was selected as the threshold level for Q3, and the thresholds for other quintiles were estimated by multiplying 40% by the ratio of average food expenditure in certain quintile to that in Q3; ^d^ Q5 = 40% means that 40% was selected as the threshold level for Q5, and the thresholds for other quintiles were estimated by multiplying 40% by the ratio of average food expenditure in certain quintile to that in Q5; ^e^ Quintile 1 is the poorest and Quintile 5 is the wealthiest.
